# Improved Charge Carrier Transport Across Grain Boundaries in N‐type PbSe by Dopant Segregation

**DOI:** 10.1002/smsc.202300299

**Published:** 2024-03-08

**Authors:** Huaide Zhang, Minghao Shen, Christian Stenz, Christian Teichrib, Riga Wu, Lisa Schäfer, Nan Lin, Yiming Zhou, Chongjian Zhou, Oana Cojocaru‐Mirédin, Matthias Wuttig, Yuan Yu

**Affiliations:** ^1^ Institute of Physics (IA) RWTH Aachen University Sommerfeldstraße 14 52074 Aachen Germany; ^2^ State Key Laboratory of Solidification Processing, and Key Laboratory of Radiation Detection Materials and Devices Ministry of Industry and Information Technology Northwestern Polytechnical University Xi'an 710072 China; ^3^ Department of Sustainable Systems Engineering (INATECH) Albert‐Ludwigs‐Universität Freiburg 79110 Freiburg Germany; ^4^ Peter Grünberg Institute (PGI 10) Forschungszentrum Jülich 52428 Jülich Germany

**Keywords:** charge carrier scattering, dopant segregations, grain boundary, lattice plainification, metavalent bonding

## Abstract

Doping is an important and routine method to tune the properties of semiconductors. Dopants accumulated at grain boundaries (GBs) can exert a profound influence on microstructures and transport properties of heat and charge. To unravel the effect of dopant accumulation at GBs on the scattering of electrons, individual high‐angle GBs in three PbSe samples doped with different amounts of Cu using a home‐designed correlative characterization platform combining electron backscatter diffraction, microcircuit transport property measurements, and atom probe tomography are studied. The findings reveal that the segregation of Cu dopants to GBs reduces the GB potential barrier height. Once the GB phase reaches an equilibrium with saturated Cu, the extra Cu dopants distribute homogeneously inside the grains, compensating for vacancies and improving the electrical conductivity of the PbSe grains. The results correlate the Cu distribution at GBs and grains with local electrical properties, enlightening strategies for manipulating advanced functional materials by GB segregation engineering.

## Graphical abstract

Grain boundary (GB) segregation affects the local microstructure, chemistry, and bonding, and thus controls the overall mechanical and functional properties of materials. With a correlative structural and transport property characterization method, it is revealed that Cu segregation to GBs in PbSe can planify the GB lattice, reducing the GB potential barrier height for charge carrier transport.

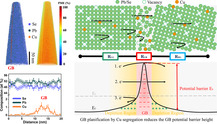

## 1 Introduction

Grain boundaries (GBs) are the interfaces between crystalline grains, which influence the transport properties of charge carriers and phonons,^[^ ^1^, ^2^ ^]^ playing a pivotal role in the materials’ design and device performance.^[^ ^3^ ^]^ In polycrystalline thermoelectric,^[^ ^4^, ^5^, ^6^, ^7^ ^]^ GBs can be used to manipulate the thermal‐to‐electricity energy conversion efficiency of materials. The performance of thermoelectric materials depends on the dimensionless figure of merit ($zT=S^{2}\sigma T/\kappa$), where *S* is the Seebeck coefficient, *σ* is the electrical conductivity, *T* is the absolute temperature, and *κ* is the total thermal conductivity.^[^ ^8^ ^]^ GBs are strategically introduced to scatter phonons for reducing thermal conductivity.^[^ ^7^ ^]^ Yet, GBs scatter also charge carriers and, thus, influence the electrical conductivity.^[^ ^9^, ^10^, ^11^, ^12^ ^]^ Normally, we need five degrees of freedom to describe a GB.^[^ ^13^ ^]^ Each of the freedoms such as the GB plane can impact the corresponding microstructures and properties of GBs.^[^ ^14^, ^15^, ^16^ ^]^ These different structures result in different GB energy and then lead to various degrees of dopant segregation.^[^ ^17^ ^]^ According to the misorientation angle, GBs are generally categorized into low‐angle GBs (LAGBs) and high‐angle GBs (HAGBs).^[^ ^1^, ^18^, ^19^ ^]^ LAGBs consist of dislocations and have a low GB energy.^[^ ^20^ ^]^ In contrast, random HAGBs show more complex and disordered atomic arrangements, which have a high GB energy and favor more dopant segregations.^[^ ^21^, ^22^, ^23^, ^24^ ^]^ It has been demonstrated in Ag‐doped PbTe that electrons will be scattered more strongly at HAGBs than at LAGBs.^[^ ^2^ ^]^ However, the influence of different segregation degrees of dopants at HAGBs on the transport of electrons has not been systematically studied. Here, the degree of segregation includes contributions from atomic arrangements and bonding mechanisms at GBs.

The complex nature of GBs leads to various phenomena in different materials. Zhang et al.^[^ ^25^ ^]^ reported on the reduction of electrical conductivity in platinum nanofilms caused by the GB charge carrier scattering. On the contrary, Carvillo et al.^[^ ^26^ ^]^ clarified that barium segregation to GBs in calcium cobaltite enhances the electrical conductivity. Villoro et al.^[^ ^27^ ^]^ and Wang et al.^[^ ^28^ ^]^ also observed increased electrical conductivity due to dopant segregation to GBs in bulk half‐Heusler alloys and SnTe thermoelectrics, respectively. However, these conclusions were achieved by measuring the transport properties averaged across different GBs and other microstructures. To unravel the impact of individual GBs on electron transport, correlative in‐depth investigations of local microstructures and transport properties of GBs are essential.

In this work, three individual HAGBs in PbSe samples doped with different amounts of Cu are studied. We focus on random HAGBs since they are ubiquitous microstructures in polycrystalline solids. We aim to elucidate the relationship between Cu distribution at GBs and the electrical properties. The Cu segregation at the GB position was identified due to the atom probe tomography (APT) capabilities (3D with a sub‐nanometer resolution).^[^ ^29^ ^]^ Moreover, APT can be combined with other techniques leading to correlative composition–properties investigations.^[^ ^2^, ^30^, ^31^ ^]^ By employing the APT‐based correlative techniques, we find that the segregation of Cu to GBs can lower the potential barrier height for electron transport due to the lattice plainification effect of Cu. This conclusion provides insights into the manipulation of electrical conductivity in polycrystalline materials.

## 2 Experimental Section

**Figure 1:**
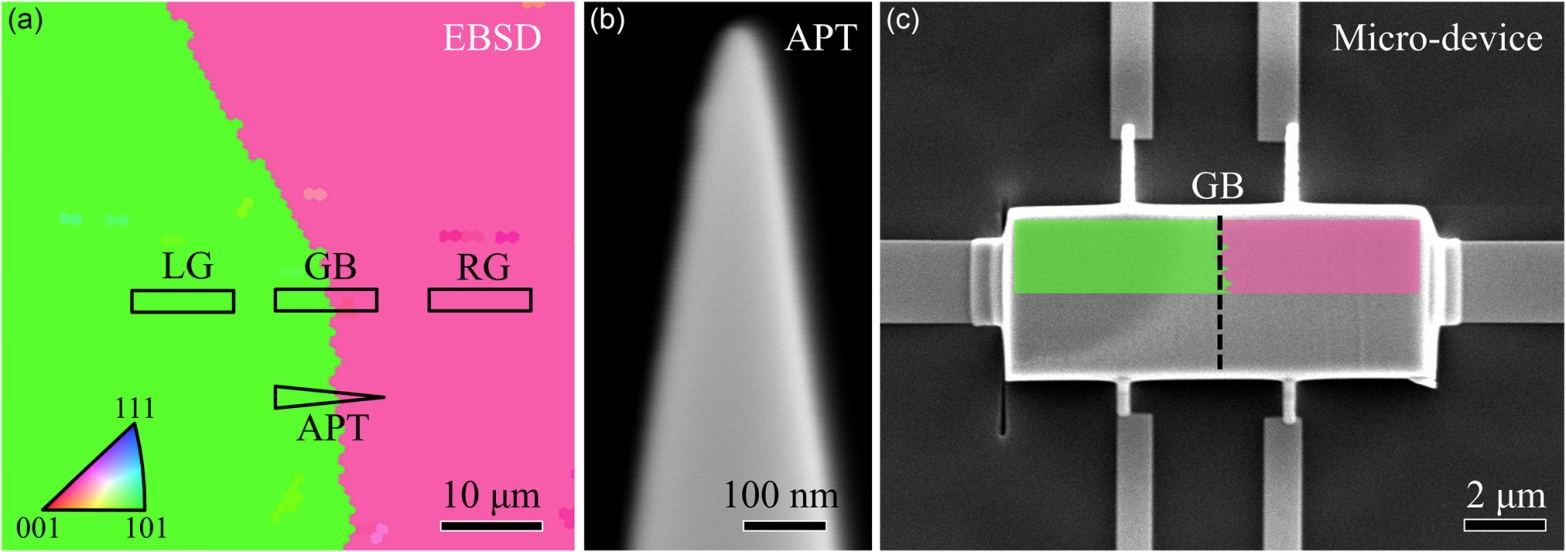
Characterization procedures for an individual GB. a) EBSD inverse pole figure map identifying a HAGB. The corresponding PPMS and APT samples are lifted out from this region. b) Scanning electron microscopy (SEM) image of the APT specimen. c) SEM image of a Hall‐bar geometry lamella containing a HAGB fixed on the microcircuit for PPMS measurement.

Copper (Cu) is a very important and efficient dopant for many thermoelectric materials.^[^ ^32^, ^33^, ^34^, ^35^ ^]^ In this work, we prepared three Cu‐doped PbSe samples with varying Cu contents of 0.005%, 0.05%, and 0.25%. The raw materials of Pb powders (99.96%, Riedel‐de Haen), Se ingots (99.99%, Strem Chemicals), and Cu granules (99.999%, Alfa Aesar) were weighed according to the above nominal compositions PbCu_*x*_Se (*x* = 0.005%, 0.05%, and 0.25%). The mixed raw materials were sealed in quartz tubes and then heated to 1273 K in 12 h and dwelled for 6 h in a tube furnace. After that, the melt was cooled down in the furnace. The obtained ingot was hand‐ground into powders with an agate mortar and pestle in an Ar‐filled glove box. The final bulk samples were obtained by compressing these powders using spark plasma sintering at 923 K for 30 min under an axial pressure of 45 MPa. An advanced correlative structure–property characterization platform developed by Wu et al.^[^ ^2^ ^]^ is utilized to study individual GBs. As illustrated in **Figure** **1**a, we first identify and locate the position of random HAGBs using electron backscatter diffraction (EBSD). Subsequently, the target HAGBs are lifted out and milled to specific shapes required for different structural and transport property characterizations using a focused ion beam (FIB, Helios NanoLab 650). Corresponding samples including GBs from samples Cu = 0.005%, Cu = 0.05%, and Cu = 0.25% are named as samples GB1, GB2, and GB3, respectively. The needle‐shaped specimens (Figure 1b) mounted on the Mo grids are prepared following the method described in the literature.^[^ ^36^ ^]^ These needles are characterized by transmission Kikuchi diffraction to confirm the position of GB and then measured by APT (LEAP 5000 XS). The experimental parameters for APT measurements include a base temperature of 40 K, a laser pulse energy of 15 pJ, a laser pulse frequency of 200 kHz, a detection rate of 1%, and a flight path of 100 mm. The APT data are processed using AP Suite 6.1. Microscale lamellae taken from the same GB and adjacent grains are lifted out with FIB and then transferred onto the microcircuits prepared by electron beam lithography and deposition. After that, the electrical properties of these lamellae are measured in a physical property measurement system (PPMS, Dynacool) from 300 K down to 50 K. The magnetic field for Hall measurements sweeps from −1 to 1 T. The GB potential barrier height was calculated using a series circuit model, as will be discussed in detail later. After obtaining the electrical transport properties and the chemical information of GBs, a microscopic understanding of the electron scattering at dopant‐decorated GBs can be established.

## 3 Results and Discussions

### 3.1 Microstructure and Composition Analysis

**Figure 2:**
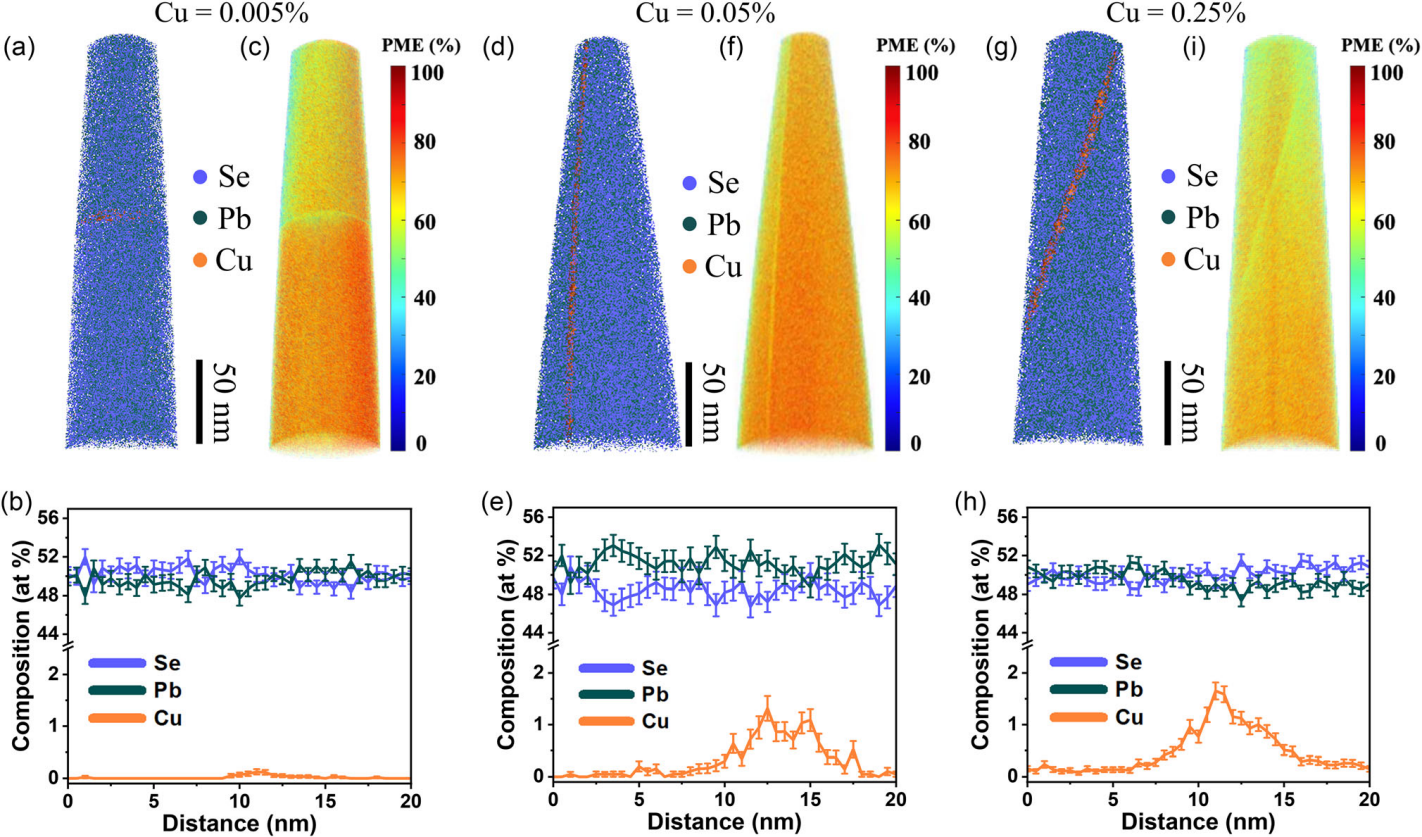
APT characterization of three specimens including one random HAGB for each taken from different Cu‐doped PbSe samples. a) 3D distribution of elements Pb, Se, and Cu in the sample with Cu = 0.005%. Slight Cu enrichment at the GB can be observed. b) 1D composition profile across the GB from top to bottom showing a maximum content of 0.15% Cu at the GB. c) PME image of the volume probed in (a), showing a large overall PME value of around 65 % in the grain interior and decreased PME in the GB. d) Elemental distribution in the sample with Cu = 0.05%. A vertical GB with enrichment of Cu can be identified. e) Composition profile across the GB showing the peak amount of Cu of around 1.5% in the GB. f) Corresponding PME image, where the GB shows a lower PME. g) Elemental distribution in the sample with Cu = 0.25%, where Cu segregation to GB can be visualized. h) Composition profile across the GB along the direction perpendicular to it, showing a maximum Cu content of 1.8 at% in the GB. i) Corresponding PME image.

To compare the effect of different dopant contents on the segregation degree and concomitant transport properties of GBs, we prepared three PbSe samples with different nominal Cu content. We first utilized APT to measure the chemical composition inside the grain interior and the GB. APT embraces a sub‐nanometer spatial resolution in three dimensions (3D) and parts‐per‐million level chemical sensitivity.^[^ ^29^, ^37^ ^]^ This enables a precise determination of the distribution and composition of trace amounts of dopants. We did not investigate pure PbSe since it is difficult to show the presence of GBs in the APT data if the GB composition is homogeneous. Moreover, the best‐performing thermoelectric materials are usually obtained by chemical doping. Since dopant segregation to GBs is inevitable, it is of paramount significance to study the impact of dopant segregation to GBs on the transport properties. The first sample contains nominally an extremely low content of Cu (0.005%). **Figure** **2**a shows the 3D distribution of constituting elements Pb, Se, and Cu as depicted by different colors of points. A careful mass spectrum analysis verifies the absence of Cu in the matrix (Figure S1, Supporting Information). In contrast, a slight enrichment of Cu at the GB1 can be observed from the local aggregated orange points. A one‐dimensional composition profile across this GB from top to bottom reveals the peak Cu content of 0.15 at% in the GB. Dopant segregation to GBs has been well described by the Gibbs adsorption isotherm,^[^ ^38^ ^]^ which was driven by the minimization of total Gibbs free energy and elastic strain energy.^[^ ^39^ ^]^ The GB segregation behavior is governed by the interactions between solutes and defects at GBs, which depends on the point defects interactions coupled with GB geometries.^[^ ^40^ ^]^ Besides the chemical composition, APT is also sensitive to bond‐breaking behavior, as can be distinguished from the values of “probability of multiple events” (PME).^[^ ^41^, ^42^, ^43^ ^]^ Figure 2c depicts the 3D PME map corresponding to the probed volume in Figure 2a. The PbSe grains show a PME value of about 65%, supporting the metavalent bonding mechanism (MVB) of PbSe, as has also been studied in previous work.^[^ ^44^, ^45^ ^]^ MVB describes a bonding situation where adjacent atoms share half an electron pair to form a sigma bond, which markedly differs from covalent, ionic, and metallic bonding.^[^ ^46^ ^]^ MVB compounds show a unique property portfolio, enabling promising applications in thermoelectrics and phase‐change memory.^[^ ^47^, ^48^, ^49^ ^]^ In contrast, the GB area shows a relatively lower PME value, possibly due to the increased local structural distortions and breakdown of MVB. It has been shown that chemical bonding is a key factor in determining solute segregation in coherent twin boundaries in Mg,^[^ ^50^ ^]^ which could also impact the segregation behavior of Cu in PbSe. A similar phenomenon has been observed in Ag‐doped PbTe.^[^ ^2^, ^9^, ^51^ ^]^ These local changes in chemical composition and bonding mechanism will influence the transport properties of electrons, as will be discussed below.

The second sample contains a higher nominal composition of Cu (0.05%). A vertical Cu‐decorated GB can be visualized in the 3D reconstruction, as shown in Figure 2d. Yet, we still do not detect Cu in the grain interior. In contrast, the content of Cu in the GB2 reaches about 1.5 at% (Figure 2e), which is about 10 times higher than that observed in the GB1, as shown in Figure 2b. Similarly, the PbSe matrix shows a high PME value due to its MVB mechanism, while the GB area shows a lower PME, as illustrated in Figure 2f. The third sample contains a considerably larger Cu composition (0.25%) than the other two samples. This also directly changes the distribution of Cu. Figure 2g shows Cu segregation to the GB3. However, the composition profile across this GB reveals the dissolution of Cu in the PbSe matrix with a composition of 0.2 at% (Figure 2h and S2, Supporting Information). In contrast, the other two samples with lower nominal Cu contents show no detectable Cu in the matrix. Interestingly, the maximum content of Cu in the GB3 does not change significantly compared to the GB2 as shown in Figure 2e. This implies a local equilibrium of the Cu content in the random HAGBs with close misorientation angles (Figure S3, Supporting Information) to form a GB complexion phase.^[^ ^52^ ^]^ The corresponding PME map shows the same phenomena as the other two samples. Note that the lower PME values on the top of APT specimens inside the grain could be induced by ion damage from sample preparation or surface contamination.

We have unraveled the distribution of Cu in the grain interior and GB in three PbSe samples with different nominal Cu contents. We will then study the electrical transport properties across exactly these same GBs to ensure a “one‐to‐one” correlation between microstructures and transport properties.

### 3.2 Electrical Transport Properties

Since GBs are two‐dimensional microstructures, it is not feasible to only measure the transport properties of GBs in a bulk sample. A Hall‐bar configuration is utilized to measure the transport properties as shown in Figure 1c. Thus, it is important to subtract the contribution from adjacent left and right grains. Therefore, we also fabricated two lamellar samples from the interior of the left and right grain relative to the GB, named LG and RG, respectively, for each Cu composition.

**Figure 3:**
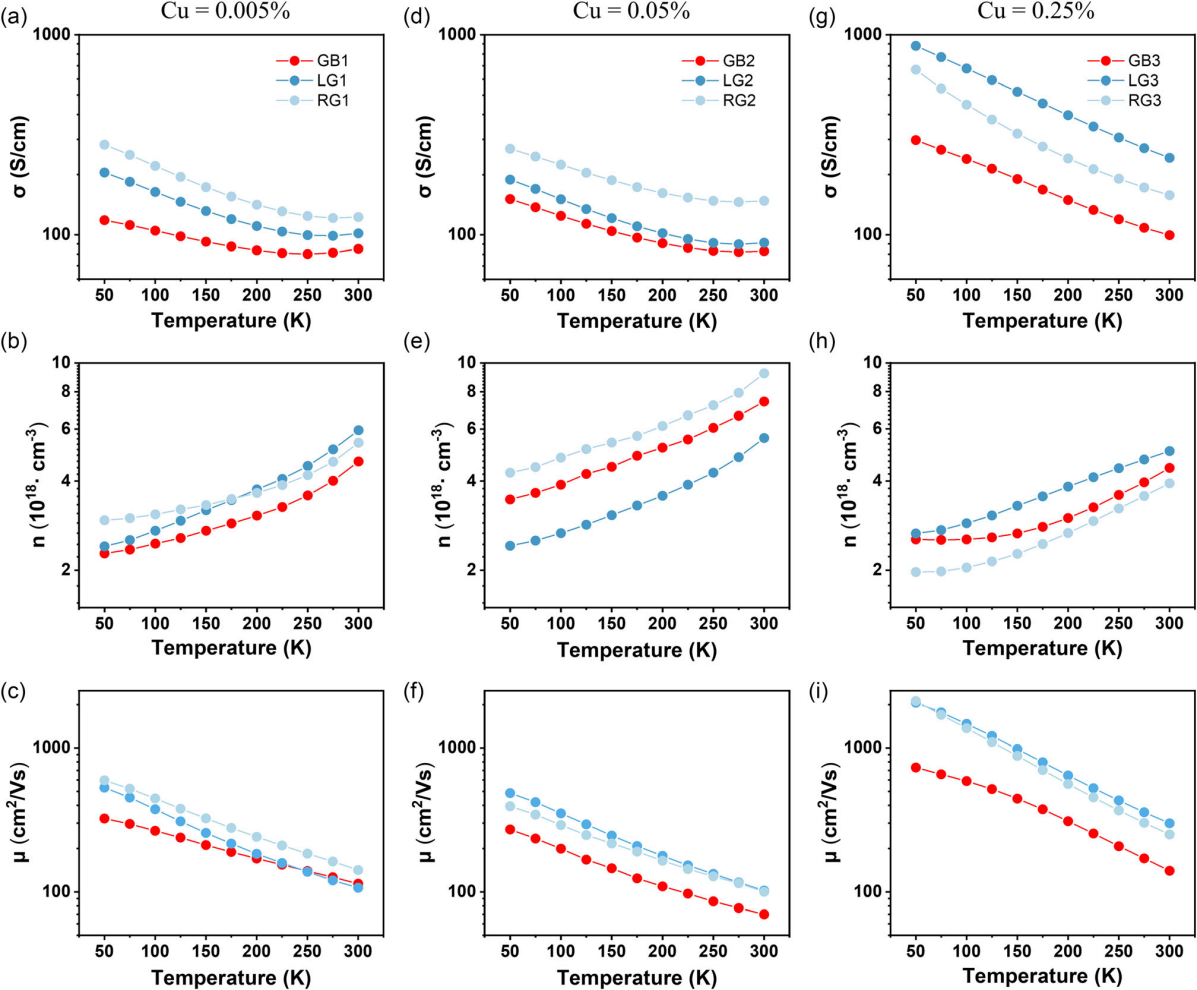
Temperature‐dependent electrical properties of lamellar samples taken from the GB area and adjacent left and right grains for three PbSe samples. a) Electrical conductivity, b) carrier concentration, and c) mobility of GB1. d) Electrical conductivity, e) carrier concentration, and f) mobility of GB2. g) Electrical conductivity, h) carrier concentration, and i) mobility of GB3. The labels of GB‐1, 2, and 3 correspond to the PbSe samples with Cu content of 0.005%, 0.05%, and 0.25%, respectively.

**Figure** **3**a shows the temperature‐dependent electrical conductivity of LG1, RG1, and GB1 lamellae taken from the sample with an extremely low Cu content of 0.005%. All three lamellae show decreased electrical conductivity with increasing temperature until 250 K, behaving as degenerate semiconductors. The upturn of electrical conductivity at higher temperatures could be induced by the intrinsic excitation given the low bandgap of PbSe (0.24 eV)^[^ ^53^ ^]^ and the ultralow content of Cu dopant. The specimen containing GB shows smaller electrical conductivity in the whole measurement temperature range. We also performed Hall measurements in the same temperature range. We observed negative Hall coefficients for all lamellar specimens, indicating n‐type conduction. Figure 3b indicates that these specimens exhibit similar values and temperature dependence of carrier concentrations regardless of the presence of GB. Heleskivi and Salo^[^ ^54^ ^]^ have demonstrated that the measured Hall carrier concentration in an inhomogeneous material consisting of low‐resistivity grains separated by high‐resistivity GBs does not differ much from that of the grains. The exponentially increased carrier concentration at high temperatures also confirms the intrinsic excitation of minority carriers. With the electrical conductivity and carrier concentration, the mobility values can be calculated, as shown in Figure 3c. The lower mobility in the GB1 specimen verifies the extra scattering source for electrons at the GB.

The lamellar specimens taken from the sample with a Cu content of 0.05% show very similar trends as observed in the previous set of specimens. The GB2 specimen still shows the lowest electrical conductivity (Figure 3d) and carrier mobility (Figure 3f) compared with its surrounding grain specimens. Yet, the variations in the electrical conductivity and carrier concentration (Figure 3e) between the LG2 and RG2 specimens seem larger than that of the previous case. This could be due to the stronger composition fluctuations of Pb and Se inside grains in this sample as characterized by APT in Figure 2e. Note that the difference in carrier concentration between LG2 and RG2 is smaller than 2 × 10^18^ cm^−3^, which can be generated by just about 0.02% Se vacancies formed during the sample preparation processes including melting, quenching, milling, and powder sintering. The different orientations of the left and right grains could also slightly impact the transport properties. The third sample with a much higher content of Cu (0.25%) still shows similar phenomena. However, the magnitude of the electrical conductivity is increased by a factor of 2–3 than the other two samples, as shown in Figure 3g. In particular, we observed a significant enhancement of the electrical conductivity in the grain interiors for samples LG3 and RG3 upon increasing the Cu content. Figure 3h shows that the carrier concentration is even slightly decreased with a higher doping content of Cu, indicating the complex occupation of Cu in the lattice that behaves differently as donors or acceptors. This also implies that the improved electrical conductivity stems not from the doping‐induced enhancement of carrier concentration, but from the elevated carrier mobility, as confirmed by Figure 3i. The specimens LG3 and RG3 show very close carrier mobility values. In contrast, the carrier mobility of specimen GB3 is significantly decreased compared with its adjacent grains. Nevertheless, the absolute mobility value of GB3 is still higher than the other two GB specimens. These different electrical transport properties among samples call for explanations from the perspective of local microstructures.

### 3.3 GB Model and Band Diagram

**Figure 4:**
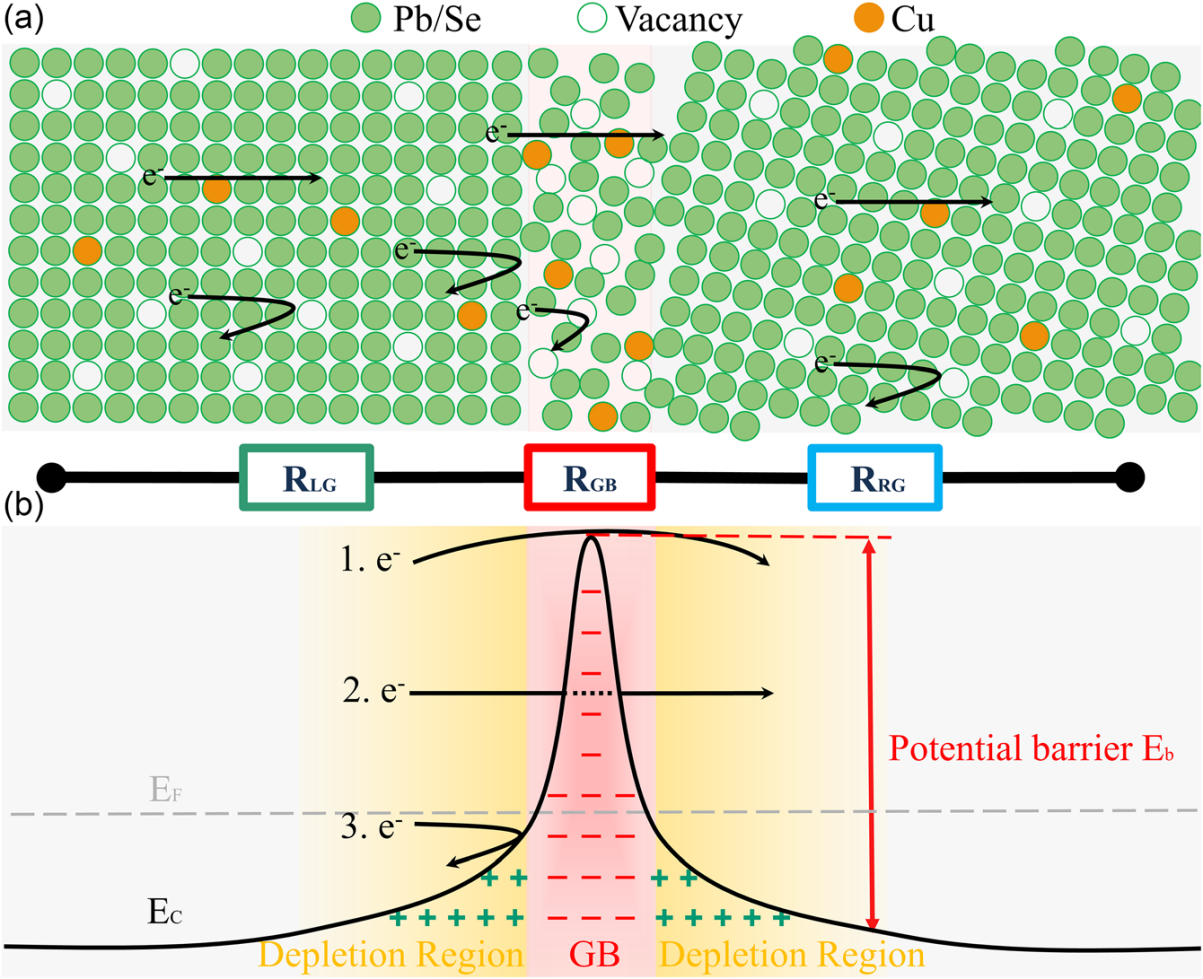
Schematic of the lattice structure with a series‐resistance model and the band diagram in the PbSe grain interiors and GB. a) The lattice structures of LG and RG exhibit distinguished orientations, resulting in the disordered structure at the interface. Cu ions are trapped easily at GBs, which could weaken the GB scattering. Some Cu ions occupy vacancies within grains enhancing the transport capability of electrons. The resistance of the lamella consists of the resistance of LG (*R*_LG_), GB (*R*_GB_), and RG (*R*_RG_). b) Band diagram in the GB regime. The GB potential barrier (*E*_b_) hinders the motion of electrons. Case 1: The electrons have enough kinetic energy to jump over the barrier. Case 2: The electrons with moderate energy have the probability of tunneling through the barrier. Case 3: The electrons have too low energy to overcome the barrier.

**Figure** **4**a schematically illustrates the atom arrangement and dopant distribution in Cu‐doped PbSe containing a HAGB. These samples were prepared by melting, quenching, and spark plasma sintering, which inherently possess imperfections such as equilibrium and nonequilibrium vacancies. The presence of GB breaks the translational periodicity of the lattice and introduces more defects in the GB. It has been demonstrated that the atomic density is about 5%–20% reduced in the GB regions compared to its adjacent grains, i.e., GBs embrace a higher amount of vacancies.^[^ ^55^ ^]^ Owing to these imperfections and disruption of chemical bonds, the GB area shows higher Gibbs free energy than the grain interior. As a consequence, dopants such as Cu prefer to segregate to GBs and vacancies to lower the overall energy as described by the Gibbs adsorption isotherm.^[^ ^56^, ^57^ ^]^ This also results in the formation of a thin layer with different compositions from the bulk, which is called GB “complexions”.^[^ ^52^ ^]^ The segregation of Cu to GBs in this work has been confirmed by APT characterizations, as shown in Figure 2. Only the sample with a high nominal Cu content (0.25%) shows observable Cu in the PbSe matrix.

We first compare the electrical transport properties for all grain specimens in Figure 2. The four lamellae taken from samples Cu = 0.005% and Cu = 0.05% show very similar electrical conductivity, carrier concentration, and mobility. APT results indicate the absence of Cu dopants in these grains, as shown in Figure 2b,e. The small variation of properties in different grains could be caused by the fluctuation of Pb and Se contents that lead to different concentrations of vacancies. In striking contrast, the sample Cu = 0.25% shows much higher electrical conductivity and carrier mobility in the grain. The major difference between this sample and the other two is the dissolution of Cu in the matrix as revealed by APT. These low content of Cu dopants in the matrix would compensate for vacancies, which is called “lattice planification” in a recent study in Cu‐doped SnSe crystal.^[^ ^58^ ^]^ Similar phenomena have also been observed in other Cu‐doped Pb chalcogenides.^[^ ^33^, ^35^, ^59^ ^]^ The lattice planification can significantly reduce the charge carrier scattering by vacancies and thus improve carrier mobility.^[^ ^58^ ^]^ Note that this conclusion is obtained by directly comparing the composition and electrical properties of single‐grain specimens in this work, which excludes other complex microstructures in bulk polycrystalline materials as much as possible.

We then compare the electrical transport properties of the specimens including individual HAGBs. All GB lamellae show lower electrical conductivity and mobility than their adjacent grain specimens, indicating extra scattering events of electrons at the GB. Yet, the measured electrical conductivity of these GB‐embedded specimens includes contributions from both the grain interior and the GB parts. Here, we introduce a series‐resistance model to explain the electrical properties of GBs. The resistance of the lamella containing a HAGB (*R*_tot_) consists of three parts, as shown in Figure 4a and Equation (1).

(1) $$R_{\text{tot}}=R_{\text{LG}}+R_{\text{RG}}+R_{\text{GB}}$$

where *R*_LG_, *R*_RG_, and *R*_GB_ are the resistance of the left grain, the right grain, and the GB, respectively. Since the electrical conductivity (resistivity) of the lamellae taken from LG, RG, and GB have been measured in Figure 3, we can derive Equation (1) as:

(2) $$\rho_{\text{tot}}\frac{L_{\text{tot}}}{wt}=\rho_{\text{LG}}\frac{L_{\text{LG}}}{wt}+\rho_{\text{RG}}\frac{L_{\text{RG}}}{wt}+\rho_{\text{GB}}\frac{L_{\text{GB}}}{wt}$$

where *w* is the width of the lamella, *t* is the thickness, *ρ* is the resistivity, and *L*_tot_ is the distance between two voltage terminals (4 μm in our case). *L*_LG_ and *L*_RG_ are the lengths of LG and RG, respectively. Since the GB is located in the middle of the lamella, and the length of the GB (*L*_GB_) is much shorter (nanometer scale) than *L*_LG_ and *L*_RG_ (micrometer scale), we assume that $L_{\text{LG}}=L_{\text{RG}}\approx \frac{1}{2}L_{\text{tot}}$. Hence, the GB interfacial resistivity 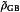 and conductivity 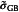 can be written as:
(3)

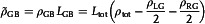



(4)

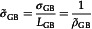

According to these equations, we can remove the contributions from the grain interiors, and calculate the GB interfacial resistivity of each HAGB. Based on the research of Ghosh et al.^[^ ^60^ ^]^ the GB interfacial conductivity can be expressed as:
(5)

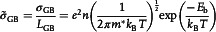

where *e* is the electron charge, *n* is the carrier concentration of GB, m* is the effective mass, *k*_B_ is the Boltzmann constant, *E*_b_ is the GB potential barrier height, and *T* is the absolute temperature.

As many parameters such as *e* and *k*_B_ are constants and other parameters such as *n* and m* are very close in the samples studied, we can derive Equation (6) from Equation (5):
(6)

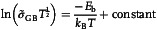

Figure 4b illustrates the band diagram of n‐type PbSe across a HAGB. Owing to the local imbalance of charges with the presence of defects, the charge transfer occurs between the GB and the grains. This results in the formation of space‐charged regions and a potential barrier that hinders the transport of carriers.^[^ ^11^, ^23^ ^]^ The electrical conductivity is contributed by the directional movement of electrons within a few $k_{B}T$ near the Fermi energy level. The high‐energy electrons have a higher probability of jumping over the barrier or across the barrier by tunneling effects. In contrast, the low‐energy electrons will be seriously scattered at the boundary. With the temperature rising, more electrons are getting enough kinetic energy to overcome the GB potential barrier. Thus, the GB scattering effect is often dominated at low temperatures. The critical temperature depends on the barrier height *E*_b_.^[^ ^23^ ^]^ Since we focus on the effect of GB microstructures on electron transport in this work, only the low‐temperature data (<200 K) will be used to fit the GB resistance and the potential barrier height. The transport properties at higher temperatures contain other contributions such as the phonon scattering and intrinsic excitations.

### 3.4 GB Interfacial Resistivity and Potential Barrier Height

**Figure 5:**
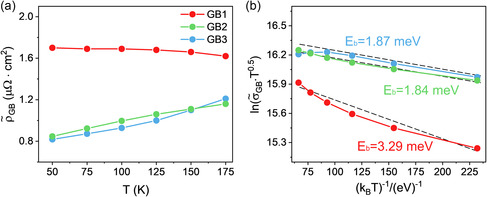
GB interfacial resistivity and the fitting of GB potential barrier height for three GB specimens investigated. a) GB interfacial resistivity calculated using Equation (3). b) Fitting of GB potential barrier height plotted as $\ln({\tilde{\sigma }}_{\text{GB}}T^{1/2})$ versus (1/*k*_B_ *T*) according to Equation (6).

**Figure** **5**a presents the GB interfacial resistivity of three HAGBs from three Cu‐doped PbSe samples calculated using Equation (3). All three specimens show positive 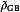, indicating that the presence of GB lowers the overall electrical conductivity. Besides the vacancy defects at GBs, the breakdown of MVB as indicated by the reduced PME value at GBs (Figure 2) can also weaken the dielectric screening effect in the GB region.^[^ ^2^ ^]^ Thus, the electrons will be subject to strong Coulomb interactions and scattered by the space charge layer around GBs. The GB1 shows an interfacial resistivity value nearly double that of the GB2 and GB3. The temperature dependence of 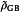 can be used to calculate the GB potential barrier height according to Equation (6). Figure 5b demonstrates that the *E*_b_ values are very close for GB2 and GB3. In contrast, the *E*_b_ for GB1 is nearly doubled.

Similar to the case of “lattice plainification” in the grain interior, the segregation of Cu to GBs can also occupy vacancies and thus plainify GBs. The GB1 contains the lowest amount of Cu as determined by APT. Therefore, there will be more remaining vacancies to scatter electrons, leading to a larger barrier height. Although the electrical conductivity and mobility in the grain interior for samples Cu = 0.05% and Cu = 0.25% are very different, their GBs show similar interfacial resistivity and potential barrier height. This is also consistent with the identical Cu composition in these two GBs. Thus, our results unambiguously reveal that Cu dopant segregation to GBs can lower the potential barrier height in PbSe. Reduced interfacial resistivity has also been found in Pb‐based chalconenides,^[^ ^61^, ^62^, ^63^ ^]^ half‐Heusler,^[^ ^27^, ^64^, ^65^ ^]^ SnTe,^[^ ^28^ ^]^ and GeTe^[^ ^66^ ^]^ alloys with the formation of conductive GB complexion phases. Nevertheless, these studies cannot exclude the contribution of other complex microstructures in bulk polycrystals. For example, the atomic structures can significantly influence the GB segregation, carrier density, and mobility. Even exactly for the same GB, the atomic structures change from site to site. Yet, from the perspective of materials design, it is reasonable to combine both the effect of GB structures and segregations. Moreover, it is also very difficult to decouple these two factors since different atomic structures influence the segregation degrees. A polycrystalline solid contains millions of various GBs. It is much more feasible to control the segregation degree rather than the atomic structures of each GB. In this regard, this work provides clear evidence of the modified interfacial resistivity upon dopant segregation by studying individual GBs, which will also in turn guide the design of materials by GB segregation engineering.

## 4 Conclusion

We studied three individual HAGBs and their adjacent grains from three Cu‐doped PbSe samples with different nominal compositions using a correlative structure–property characterization platform. We found that GBs are preferential sites for the segregation of Cu dopants. The content of Cu in the GB increases from 0.15 at% in the sample Cu = 0.005% to 1.5 at% in the sample Cu = 0.05%. Further increasing the nominal Cu content to 0.25% does not significantly increase the Cu concentration in the GB but influences the composition of the grain interior. The dissolution of Cu in the PbSe matrix plainifies the lattice by filling up vacancies. Thus, the electrical conductivity and mobility inside the grain are greatly enhanced. The presence of GB lowers the overall electrical conductivity due to structural defects and the collapse of metavalent bonding at GBs. Different segregation degrees of Cu to GBs influence the electron transport behavior. A higher content of Cu at the GB reduces the potential barrier height. This research offers valuable insights into the manipulation of charge carrier transport properties in thermoelectric materials and other semiconductors. The findings pave the way for enhanced thermoelectric performance through controlled doping and GB engineering, making it a promising avenue for materials design and development.

## Conflict of Interest

The authors declare no conflict of interest.

## Supporting information

Supplementary Material

## Data Availability

The data that support the findings of this study are available from the corresponding author upon reasonable request.
