# Supplementary material for: Improved Charge Carrier Transport Across Grain Boundaries in N‐type PbSe by Dopant Segregation
Source: Small Sci. 2024 Mar 8;5(3):2300299. doi: 10.1002/smsc.202300299 (PMC12245035; doi:10.1002/smsc.202300299)
Supplement: Supplementary file 1 — Supplementary Material [file SMSC-5-2300299-s001.pdf]

Supporting Information

Improved charge carrier transport across grain boundaries in n-type PbSe by dopant segregation

Huaide Zhang,[1] Minghao Shen,[1] Christian Stenz,[1] Christian Teichrib,[1] Riga Wu,[1] Lisa Schäfer,[1] Nan Lin,[1] Yiming Zhou,[1] Chongjian Zhou,[2] Oana Cojocaru-Mirédin,[3] Matthias Wuttig,[1,4] and Yuan Yu[1*]

[1] Institute of Physics (IA), RWTH Aachen University, Sommerfeldstraße 14, 52074 Aachen, Germany

[2] State Key Laboratory of Solidification Processing, and Key Laboratory of Radiation Detection Materials and Devices, Ministry of Industry and Information Technology,

Northwestern Polytechnical University, Xi'an, 710072, China

[3] Department of Sustainable Systems Engineering (INATECH), Albert-Ludwigs-Universität Freiburg, 79110 Freiburg, Germany

[4] Peter Grünberg Institute (PGI 10), Forschungszentrum Jülich, 52428 Jülich, Germany

[*] Corresponding author: yu@physik.rwth-aachen.de

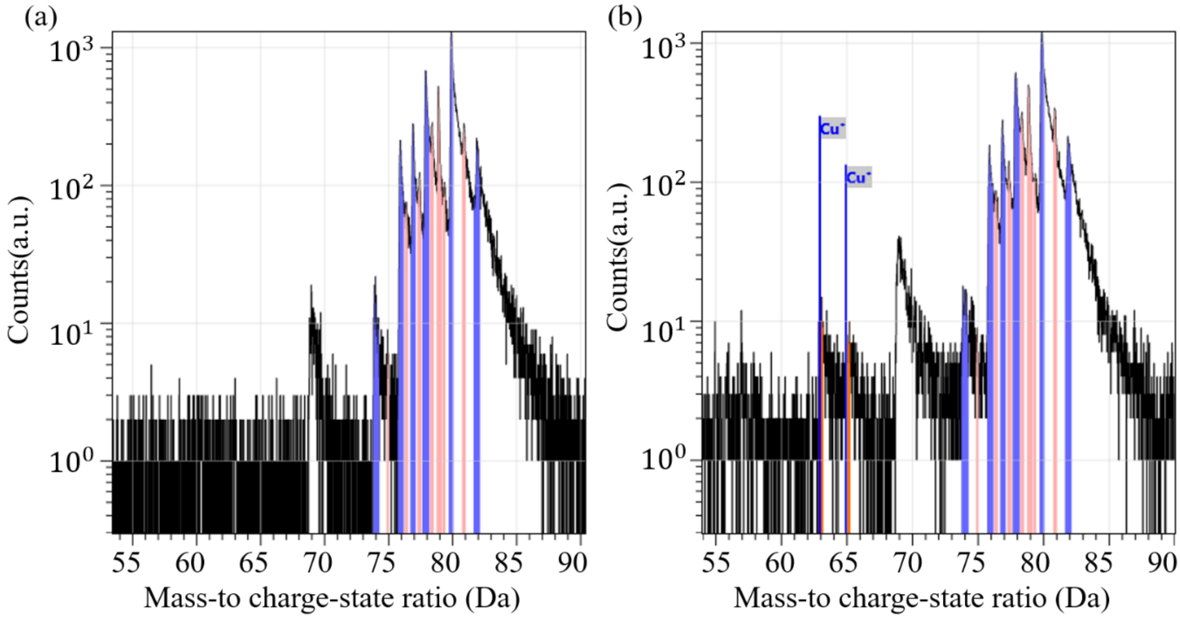

Figure S1: Mass spectrum of the PbSe specimen with Cu=0.005%, (a) in the matrix, (b) across GB.

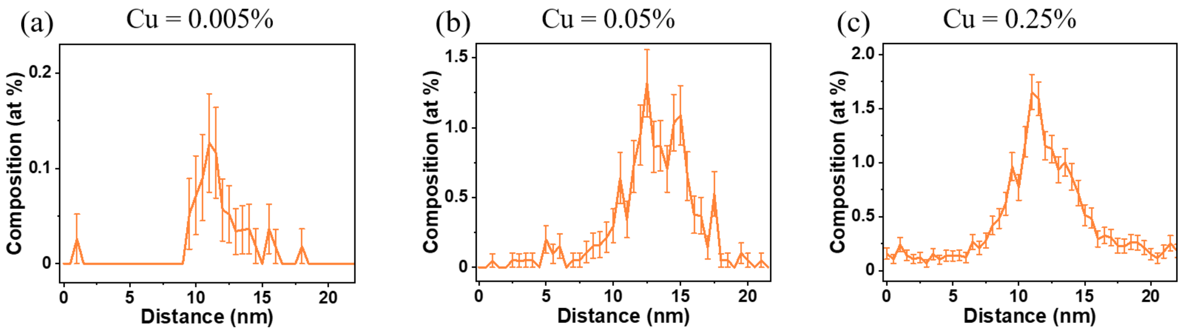

Figure S2: Copper (Cu) composition in the GB regions of PbSe with Cu= (a) 0.005%, (b) 0.05%, and (c) 0.25%.

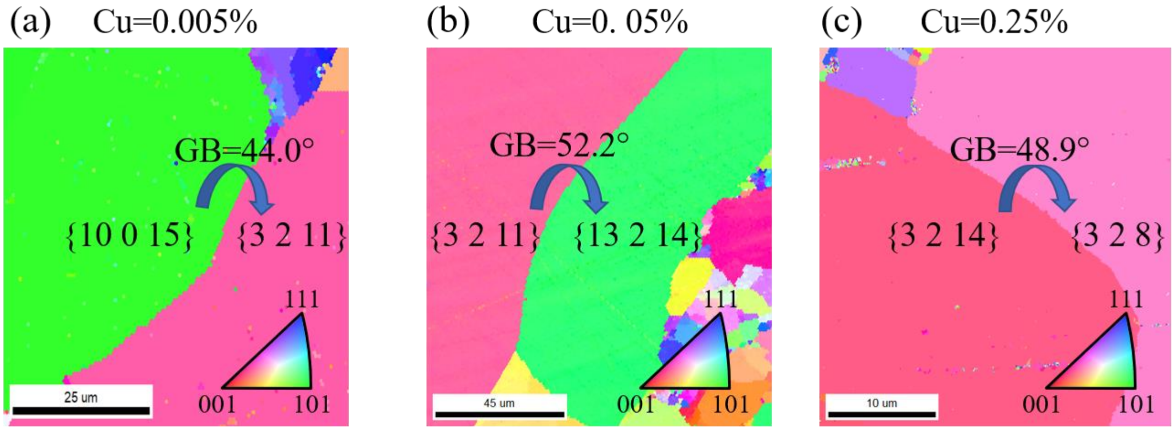

Figure S3: The EBSD maps in the GB regime belonging to three Cu-doped PbSe solids. (a) PbSe with Cu=0.005%, the angle of GB is 44.0° ({10 0 15}/{3 2 11}), (b) PbSe with Cu=0.05%, the angle of GB is 52.2° ({3 2 11}/{13 2 14}), (c) PbSe with Cu=0.25%, the angle of GB is 48.9° ({3 2 14}/{3 2 8}).
